# “I’m Not Alone on This Journey—There Are Good People Out There”: Social Connectedness Among People With LTSS Needs

**DOI:** 10.1093/geroni/igaf122.3875

**Published:** 2025-12-31

**Authors:** Sarah Sunghye Kang, Jianan Li, Lené Levy-Storms, Kathryn Kietzman

**Affiliations:** University of California, Los Angeles, Los Angeles, California, United States; University of California, Los Angeles, Los Angeles, California, United States; UCLA Luskin School of Public Affairs/Social Welfare, Los Angeles, California, United States; UCLA Fielding School of Public Health, Los Angeles, California, United States

## Abstract

The demand for Long-Term Services and Supports (LTSS) in California continues to rise, with an estimated 4.1 million adults living with disabilities or chronic conditions. People with LTSS needs often experience physical isolation, which reduces opportunities to build and maintain social relationships and stay engaged in society. At the same time, social connections support well-being; yet, the lived experiences of social connectedness among this population remain understudied. The California LTSS Study includes a qualitative component that explores the experiences of older adults and adults who have difficulties with activities of daily living. We conducted open-ended, in-person interviews with a purposive sample of 90 participants. Preliminary analyses with a subgroup of 12 participants revealed insights about their experiences of social isolation and connectedness. Using analytical approaches guided by constructivist grounded theory, we identified three categories describing the lived experiences of social connectedness among people with LTSS needs: 1) the sources of social connections (e.g., family and friends, cohabitants, caregivers, community involvement, and employment), 2) perceived changes in social connections (e.g., COVID-19, worsening health conditions, and loss or gain of loved ones or caregivers), and 3) barriers and bridges to social connections (e.g., technology, mobility, accessibility, social perceptions of disability, and internal resources). These preliminary findings characterize aspects of social connectedness using the voices of people with LTSS needs and suggest the need for more in-depth, qualitative analyses that examine both opportunities and challenges related to the social well-being of this vulnerable population.

